# Enhancing Oral Cancer Detection: A Systematic Review of the Diagnostic Accuracy and Future Integration of Optical Coherence Tomography with Artificial Intelligence

**DOI:** 10.3390/jcm13195822

**Published:** 2024-09-29

**Authors:** Waseem Jerjes, Harvey Stevenson, Daniele Ramsay, Zaid Hamdoon

**Affiliations:** 1 Research and Development Unit, Hammersmith and Fulham Primary Care Network, London W6 7HY, UK; 2 Faculty of Medicine, Imperial College London, London W12 0BZ, UK; harvey.stevenson18@imperial.ac.uk (H.S.); daniele.ramsay18@imperial.ac.uk (D.R.); 3 Department of Oral and Craniofacial Health Sciences, College of Dental Medicine, University of Sharjah, Sharjah P.O. Box 27272, United Arab Emirates; zothman@sharjah.ac.ae

**Keywords:** OCT, oral cancer, diagnostic accuracy, artificial intelligence, imaging modalities

## Abstract

**Introduction**: Optical Coherence Tomography (OCT) has emerged as an important imaging modality in non-invasive diagnosis for oral cancer and can provide real-time visualisation of tissue morphology with the required high resolution. This systematic review aims to assess the diagnostic accuracy of OCT in the detection of oral cancers, and to explore the potential integration of OCT with artificial intelligence (AI) and other imaging techniques to enhance diagnostic precision and clinical outcomes in oral healthcare. **Methods**: A systematic literature search was conducted across PubMed, Embase, Scopus, Google Scholar, Cochrane Central Register, and Web of Science from inception until August 2024. Studies were included if they employed OCT for oral cancer detection, reported diagnostic outcomes, such as sensitivity and specificity, and were conducted on human subjects. Data extraction and quality assessment were performed independently by two reviewers. The synthesis highlights advancements in OCT technology, including AI-enhanced interpretations. **Results**: A total of 9 studies met the inclusion criteria, encompassing a total of 860 events (cancer detections). The studies spanned from 2008 to 2022 and utilised various OCT techniques, including clinician-based, algorithm-based, and AI-driven interpretations. The findings indicate OCT’s high diagnostic accuracy, with sensitivity ranging from 75% to 100% and specificity from 71% to 100%. AI-augmented OCT interpretations demonstrated the highest accuracy, emphasising OCT’s potential in early cancer detection and precision in guiding surgical interventions. **Conclusions**: OCT could play a very prominent role as a new diagnostic tool for oral cancer, with very high sensitivity and specificity. Future research pointed towards integrating OCT with other imaging methods and AI systems in providing better accuracy of diagnoses, plus more clinical usability. Further development and validation with large-scale multicentre trials is imperative for the realisation of this potential in changing the way we practice oral healthcare.

## 1. Introduction

Optical Coherence Tomography (OCT) has been used to provide a significant advancement in medical imaging, particularly for its use in oral tissues [1]. OCT technology, based on low-coherence interferometry, allows the capture of real-time, high-resolution (on the order of microns) imaging of biological tissues. For the first time, an unparalleled visualisation of oral tissue microstructures was made without any invasive procedure [2,3,4]. Such a non-invasive modality has emerged to be an exceptionally versatile technique that has broadened its application in nearly all medical practices, with oral tissue diagnostics being a modality whose effect OCT has exceedingly succeeded in changing [3,4,5,6].

### 1.1. Types and Advantages of OCT

Two primary OCT types have emerged: time-domain OCT (TD-OCT) and frequency-domain OCT (FD-OCT), which includes spectral-domain OCT (SD-OCT) and swept-source OCT (SS-OCT) [1,2]. TD-OCT captures depth information sequentially, while FD-OCT captures all depth information simultaneously, greatly increasing the image acquisition speed [2]. SS-OCT achieves faster scanning speeds and deeper tissue penetration [2,3,7,8]. Advantages of OCT over conventional imaging procedures are plenty. Its safety in terms of a non-ionised radiation source is coupled with the potential for real-time imaging, thereby placing it as a superior diagnostic tool and more to the benefits accrued in the early detection and management of oral diseases [1]. All these qualities, accompanied by the latest progress in the portable and handheld design of OCT systems, make this technology on the brink of revolutionising the diagnostic practice, with substantial changes in patient outcomes and quality of care [7,9].

### 1.2. Principles and Techniques of OCT

**Figure 1:**
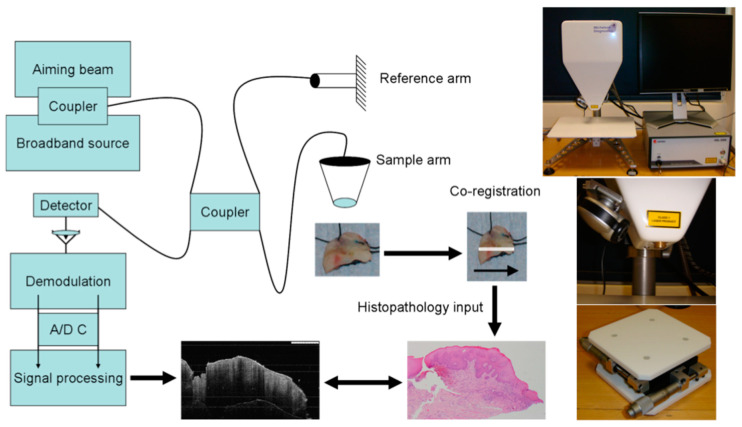
Schematic diagram illustrating the co-registration technique between the OCT machine and the tissue sample. Top right: Swept-source Fourier-domain OCT device (Michelson Diagnostics) for imaging. Middle right: The sample arm equipped with a scanning laser and digital camera for precise imaging. Bottom right: Manually adjustable stage to optimize the tissue sample’s position during scanning.

OCT uses light-wave interference from a low-coherence interferometer that captures high-resolution, cross-sectional images of biological tissues [10,11,12,13]. OCT is based on a low-coherence interferometer that relies on a special setup of a broadband light source, separation of a reflected light into reference and sample beams, and recombination to create an interference pattern—offering elaborated images of the tissue with resolution in the order of micrometres (Figure 1) [14,15,16,17]. Near-infrared light allows OCT to penetrate the tissues at a depth of 2–3 mm, which is very much helpful in the assessment of oral pathologies that are not visible from the surface [16,18,19,20,21,22]. OCT offers potential in vivo imaging with high resolution and an imaging capability that can greatly assist clinicians through real-time imaging in making informed decisions related to diagnosis and treatment [4,8,23]. It is non-invasive, avoiding any discomfort to the patients, as well as any risk of complications with invasive procedures [24,25,26,27,28,29,30]. Although OCT is a very useful imaging technique, it is prone to sensitivity from motion and, therefore, the patient must remain still during imaging, and the penetration depth may be inadequate to visualise deeper pathologies [7,11,25,26,31,32,33,34,35,36].

### 1.3. Applications of OCT in Oral Health

OCT has revolutionised early dental caries detection and monitoring, both in its diagnosis and preventive management, which is a common issue that affects dental health globally. Traditional methods for the detection of caries, including visual inspection and radiography, are typically inefficient in the early detection of demineralisation and subsurface lesions, which is key to preventive care. OCT’s high-resolution imaging uncovers initial signs of decay and provides detailed visualisations of the structure of the tooth, pinpointing subtle changes in enamel, which are characteristic of initial caries development. In this respect, OCT is used in the diagnosis of occlusal and interproximal caries: determination of the progression of a carious lesion and guiding its treatment through monitoring dynamic changes inside tooth structures, determining lesion activity, and tailoring minimally invasive treatment strategies. The promise of OCT has been demonstrated to be in non-invasive monitoring of remineralisation strategies and therapeutic interventions, which monitor the changes in tooth structure without causing harm to patients, thus refining treatment plans based on the feedback in real time [37,38]. Some of the other potential barriers for integration of OCT into the mainstream for caries management include issues such as the need for standardised image interpretation protocols and comprehensive clinician training to discern subtle changes in optical properties of tooth tissues against a background of artifacts and noise [39].

For the diagnosis and treatment of periodontal disease, OCT has become an indispensable tool in visualising conditions that affect large populations worldwide by causing inflammation and destruction of tooth-supporting structures. These improved resolutions will assist in visualising periodontal issues, such as subgingival calculus, pocket depth, and root surface condition. This improved detection capacity then influences the prognosis and treatment pathway selection. OCT discriminates the structural changes between healthy and diseased tissues, resulting in a shift in their reflectivity properties, thus providing valuable information for both accurate monitoring and management of periodontal disease progression [40,41]. Longitudinal monitoring has been based on follow-up with OCT and has been able to trace disease progression, intervention effectiveness, and minimally invasive, patient-friendly methods that reduce discomfort and the risk of tissue damage and increase patient compliance. These findings empower OCT with the power of strategic planning on periodontal interventions and act as an educational tool that empowers the patient with visual understanding of their condition and the need for maintaining periodontal health [42,43,44].

Consequently, the OCT technique is capable of producing high-resolution, cross-sectional images that allow the study of the tissue architecture. It assists in differentiating between benign and potentially malignant conditions through the evaluation of optical properties and microstructures within the lesions; thus, it provides a clear vision of layered oral mucosa and submucosal features that are critical in treatment planning and ensuring safety to the patient [45]. This significantly widens the application of OCT from initial diagnosis to following up lesion progression or regression over time without invasive biopsies, which is very helpful for conservative management of the lesions and close follow-up with timely interventions. In a post-treatment scenario, OCT identifies a recurrence early and allows for an effective treatment plan. OCT is a non-invasive technique that is beneficial for paediatric and anxious patients, thus offering a comfortable diagnostic approach and ensuring adherence to the treatment plan and regular follow-ups. This is especially important for lesions with the potential for malignancy [46,47,48].

**Figure 2:**
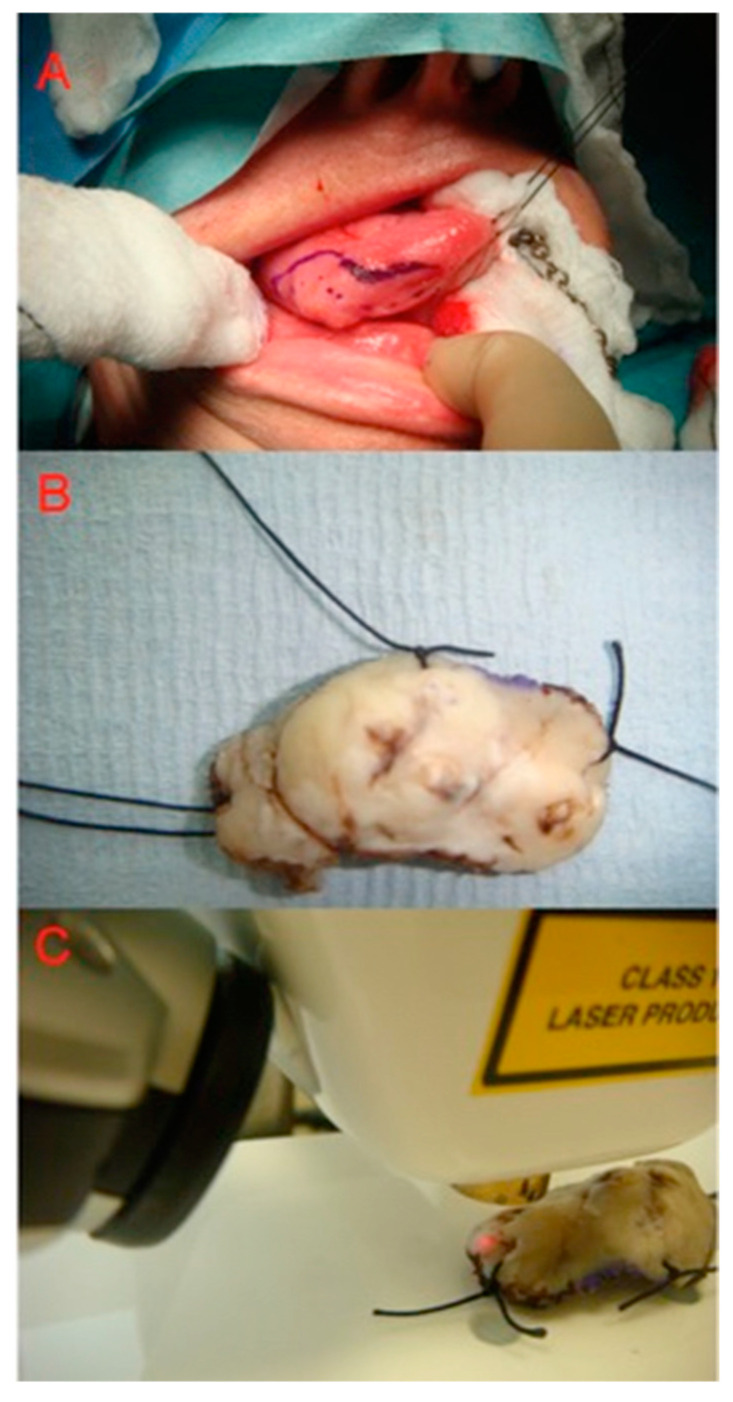
(**A**) Lateral tongue SCC prior to resection, (**B**) sutures and dye applied to improve the process of co-localisation, and (**C**) specimen scanned with OCT.

**Figure 3:**
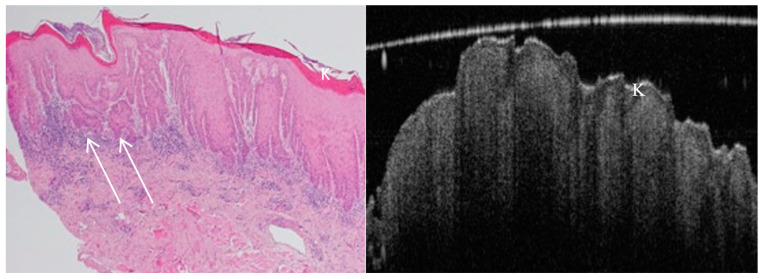
In vivo OCT and histopathology images reveal hyperkeratosis and severe epithelial dysplasia (K) of a speckled leukoplakia on the floor of the mouth. Early invasive carcinoma (arrows) shown on histology cannot be visualised on the corresponding OCT image due to the lack of sufficient depth of penetration as well as inferior image resolution compared to the H&E slide.

OCT is important for the detection and monitoring of pre-malignant lesions, rendering this stage rather critical for invasive oral squamous cell carcinoma (OSCC; Figure 2). It records minute architectural changes within tissues before malignancy, which can hardly be detected with routine clinical inspections. Serial assessment with OCT monitors lesion progression over time, therapeutic response, and the need for additional interventions to halt the development of OSCC [1,5]. The diagnostic accuracy with OCT is refined by the visualisation of microstructural changes indicative of dysplasia, such as variations in epithelial thickness, basement membrane integrity, and vascular patterns (Figure 3). All of these facilitate differentiation between benign, dysplastic, and malignant tissues to ensure an accurate biopsy sample that targets the appropriate representative lesion sites to be sent for histopathological analysis. The value of OCT in diagnostics and management strategies for pre-malignant conditions is further underscored by the fact that targeted biopsy guidance, coupled with OCT, is valuable in ongoing treatment monitoring and lesion surveillance [6,8,49,50].

**Figure 4:**
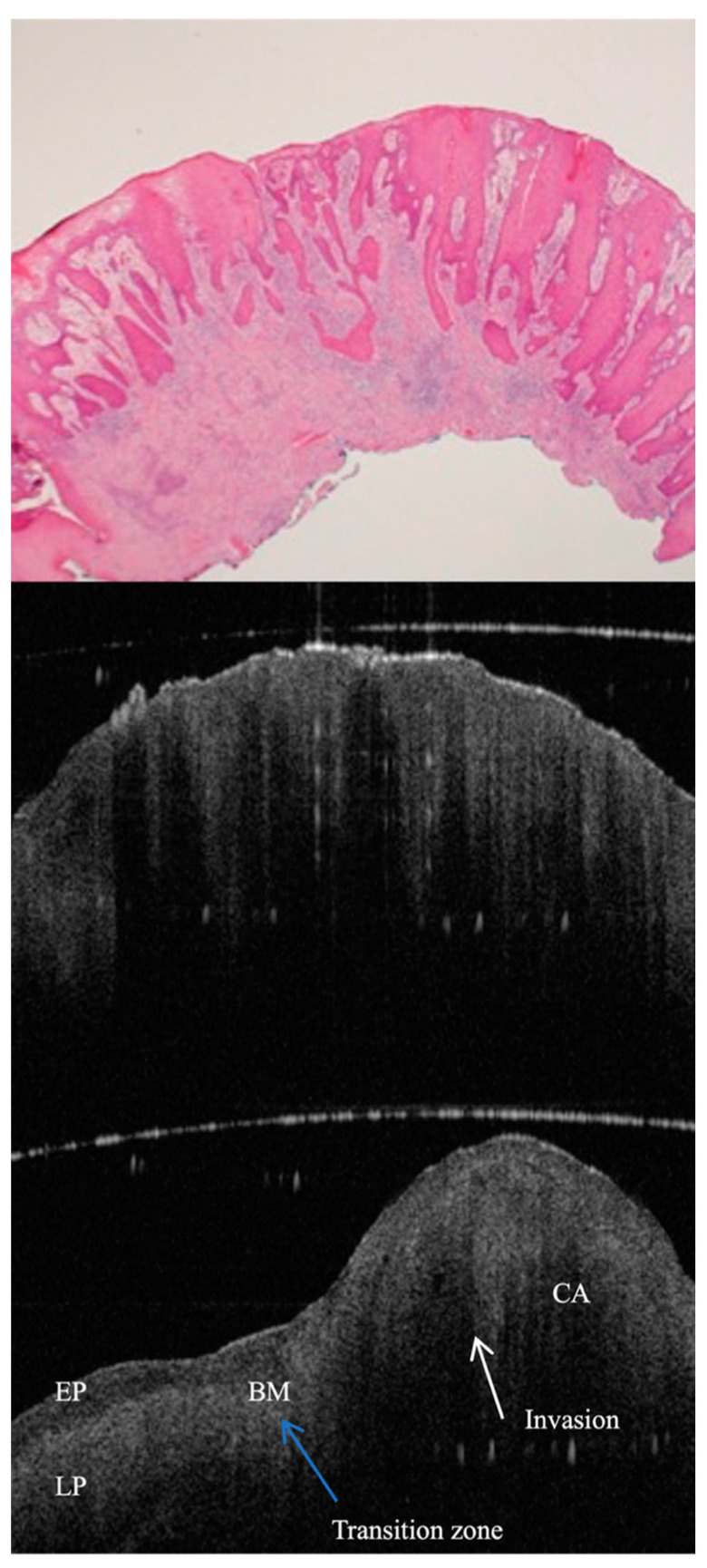
In vivo OCT and histopathology images (**top** and **middle**) of an erythro-leukoplakic lesion on the soft palate, revealing multifocal carcinoma (CA). OCT image matches histopathology in displaying multifocal epithelial downgrowth and invasion into the subepithelial layers (white arrows). Furthermore, the basement membrane is indiscernible through the entire OCT scan as a coherent prominent landmark. The bottom image is an in vivo OCT image of a transition zone (blue arrow) between healthy tissue and invasive carcinoma (CA), invading through the basement membrane. It also shows normal-thickness-stratified squamous epithelium, which is darker compared to the homogeneous lamina propria. While crossing the transition zone, epithelial downgrowth and invasion can be clearly recognised, and lamina propria become non-homogeneous. BM = basement membrane; EP = epithelium; LP = lamina propria; CA = invasive carcinoma.

The OCT can sense early and subtle morphological alterations in the oral mucosa not appreciable by visual examination. This places it as a key tool in early detection and management of OSCC. High-resolution images penetrate 2 to 3 mm beneath the tissue surface, which is crucial for early cancer detection. The keratin layer, basement membrane integrity, and vascularisation are all tissue irregularities that, when defined within the OCT image, determine the importance of OCT in the early diagnosis of oral cancers, which will significantly improve patient outcomes (Figure 4) [1,5,7,8,9,15,49,50,51]. The accuracy of real-time imaging by OCT is particularly important during surgical interventions, where it can be used to delineate accurate tumour margins to ensure the removal of all malignant tissues and yet leave all healthy tissues that are vitally important for the functions and aesthetics of the oral cavity. Following treatment, the OCT method is further applied to monitor treatment efficacy, detection of residual disease, and identification of signs of recurrence in a non-invasive manner, allowing continued patient monitoring without creating any discomfort, so that clinicians get an additional tool for evaluating and adapting treatment plans, further illustrating how OCT becomes invaluable in the context of comprehensive management and follow-up care of oral cancer [52,53].

OCT goes far beyond the conventional imaging and provides information on a variety of oral and maxillofacial conditions non-invasively. In salivary gland pathologies, including conditions such as Sjögren’s syndrome and salivary gland tumours, OCT imaging shows ductal architecture and parenchyma for early abnormality detection. It increases the pace of diagnosis and treatment onset, with better outcomes for the patient. Applications of OCT extend to the TMJ, through the identification of early degenerative changes within the cartilage and synovial fluid, and proactive management of disorders of the TMJ, by timely interventions [54,55]. OCT’s versatility has been proven in the field of dental restoration, orthodontics, and endodontics, whereby it provides non-destructive ways of testing the integrity of restorations, monitoring tooth movement, and supporting structures’ health, in addition to the evaluation of dental pulp health, identification of fractures, and measurement of dentin thickness, allowing for preservation of tooth vitality and prevention of complications [56,57,58].

### 1.4. Study Aim

This systematic review aims to assess the diagnostic accuracy of Optical Coherence Tomography (OCT) in detecting oral cancers, and to explore the potential integration of OCT with artificial intelligence (AI) and other imaging techniques to enhance diagnostic precision and clinical outcomes in oral healthcare.

## 2. Materials and Methods

This systematic review (PROSPERO registration: CRD42024593170) was conducted and reported in accordance with the Preferred Reporting Items for Systematic Reviews and Meta-Analyses (PRISMA) guidelines to ensure comprehensive and transparent reporting. A comprehensive literature search was executed on 1 December 2023 and repeated on 1 September 2024 to ensure the inclusion of contemporary evidence across several databases, including PubMed, Embase, Scopus, and Web of Science, from their inception until August 2024. The search strategy incorporated terms related to “optical coherence tomography”, “oral premalignancy”, “oral precancer”, “oral dysplasia”, “oral cancer”, “oral malignancy”, and “oral carcinoma”. Reference lists of identified studies were manually searched to ensure comprehensive coverage. Studies were included if they utilised OCT for oral cancer detection, reported diagnostic outcomes, such as sensitivity and specificity, and were conducted on human subjects. Exclusion criteria were reviews, editorials, and case reports/series, studies not reporting specific diagnostic outcomes, non-English language studies, and the study selection process.

**Figure 5:**
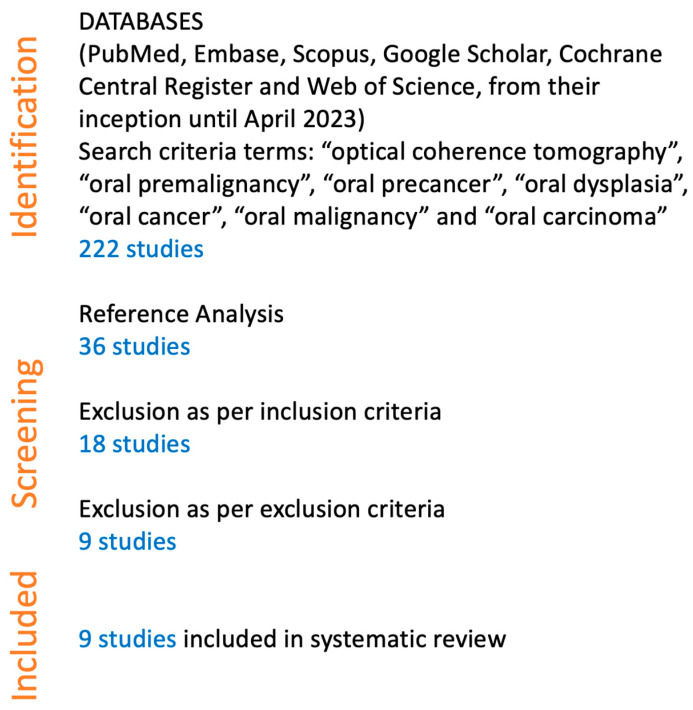
Flow chart illustrating the search protocol.

After removing duplicates, 222 studies remained for screening. Of these, 36 studies were selected for reference analysis. Based on the inclusion criteria, 18 studies were excluded. A further 9 studies were excluded based on the exclusion criteria. Ultimately, 9 studies were included in the systematic review (Figure 5).

Two independent reviewers (DR and HS) screened the titles and abstracts of the retrieved records for eligibility. Discrepancies were resolved through discussion by consulting a third reviewer (WJ). The full texts of potentially eligible articles were then assessed for inclusion, using the predetermined criteria. Data were extracted by two reviewers independently using a standardised form, including study characteristics (author, year, and country), participant demographics, OCT technology and methodology (device and image interpretation technique), and diagnostic accuracy measures (sensitivity, specificity, and total sample size).

Initially, a meta-analysis was planned to aggregate sensitivity and specificity data across studies. However, due to the heterogeneity in OCT techniques, interpretation methods, and study populations, a meta-analysis was deemed inappropriate. Heterogeneity was evident in the technological aspects of OCT devices, the clinical settings in which they were used, and the criteria for interpreting OCT images. The studies utilised various OCT technologies, ranging from earlier versions of the technology to advanced AI-driven interpretations, making direct comparison challenging.

Instead of a meta-analysis, a qualitative synthesis was conducted. This approach allowed for a detailed examination of the individual studies, highlighting the evolution of OCT technology in oral cancer detection and the impact of different image interpretation methods on diagnostic accuracy. This review did not require ethical approval, as it involved the synthesis of published data without directly involving human participants or accessing individual patient data.

Quality assessment of the studies was conducted using the Risk of Bias in Non-Randomised Studies—of Interventions (ROBINS-I) tool. This assessment critically evaluated bias across seven domains: confounding, selection of participants, classification of interventions, deviations from intended interventions, missing data, measurement of outcomes, and selection of reported results. Each study was examined for biases that could potentially influence the study outcomes, focusing on the methodological quality and the transparency of reporting the results. The risk of bias was classified as low, moderate, high, or critical for each domain based on predefined criteria. This systematic approach ensures a rigorous and unbiased synthesis of the evidence regarding the utility of OCT in the diagnosis and management of oral cancer.

Effect measures for the outcomes included sensitivity and specificity, which were the primary diagnostic accuracy metrics used in this review. These measures were chosen to facilitate a consistent evaluation of OCT’s diagnostic performance across different studies.

## 3. Results

The search and selection process identified nine studies that met the inclusion criteria, as detailed in the PRISMA flow diagram (Figure 5). Data were extracted from the 9 studies, with 860 events (cancer detections) reported. The included studies spanned from 2008 to 2022 and employed various OCT techniques, including clinician-based, algorithm-based, and artificial intelligence (AI)-driven interpretations.

The nine studies [5,19,21,28,52,59,60,61,62] reviewed highlighted the development and refinement of OCT systems, including swept-source and handheld devices with automated diagnostic algorithms, and their roles in image acquisition, interpretation, and the clinical relevance of these advancements. OCT’s high-resolution imaging capabilities allow for non-invasive, real-time assessments of oral tissue architecture, providing valuable insights into epithelial thickness, tissue texture, and other morphological changes associated with oral pathology. The importance of OCT in oral pathology assessment is underscored by its high sensitivity and specificity in identifying malignancies, delineating surgical margins, and differentiating between normal, dysplastic, and cancerous tissues. The integration of OCT with AI and machine learning algorithms further enhances its diagnostic accuracy, making it a promising tool for early detection, guided biopsy, and treatment planning in oral cancer management (Table 1).

**Figure 6:**
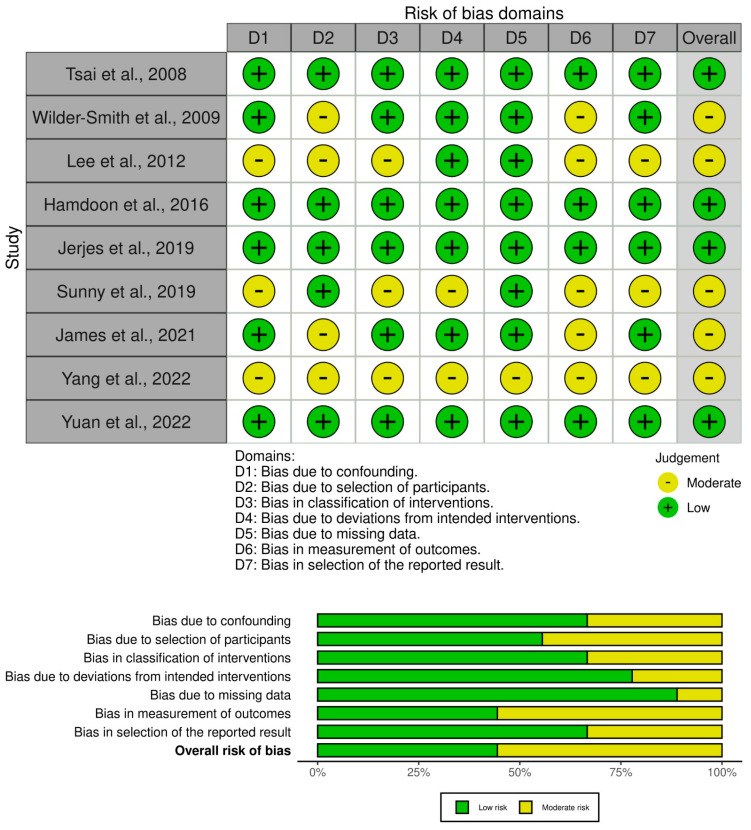
Summary plots demonstrating the risk-of-bias assessment using the ROBINS-I tool for non-randomised studies. Most studies were found to have a moderate risk of bias, 5/9. The remaining four studies were found to have a low risk of bias [5,19,21,28,52,59,60,61,62].

The overall risk-of-bias assessment for the nine studies evaluated ranges from low to moderate (Figure 6), indicating a generally reliable body of evidence but with certain limitations, particularly in the selection of participants, measurement of outcomes, and classification of interventions in some studies. These limitations highlight the need for careful consideration of study designs in future research on the diagnostic applications of OCT in oral cancer.

## 4. Results of Individual Studies

Five studies employed clinician-based interpretations of OCT images. These studies demonstrated a range of sensitivity from 75% to 92% and specificity from 71% to 93%. Tsai et al. [59] reported sensitivity and specificity of 75% and 71%, respectively, using a swept-source OCT system to analyse indicators, such as standard deviation, the decay constant, and epithelium thickness. Wilder-Smith et al. [60] achieved higher diagnostic accuracy, with sensitivity and specificity of 91% and 93%, respectively, highlighting the capability of OCT in detecting oral premalignancy and malignancy. Lee et al. [61], Hamdoon et al. [5], and Jerjes et al. [21] further corroborated the utility of OCT, reporting sensitivity and specificity within the ranges mentioned, emphasising the role of epithelial thickness and architectural changes in diagnosing oral cancer (Table 2).

Sunny et al. [28] and James et al. [62] explored the use of algorithm-based diagnostic platforms for OCT image interpretation. Both studies showcased excellent results, with Sunny et al. [28] achieving perfect sensitivity and specificity of 100%. James et al. [62] reported sensitivity and specificity of 95% and 76%, respectively, validating the efficacy of a portable OCT device combined with a machine learning algorithm in different clinical settings (Table 2).

The most recent studies by Yang et al. [19] and Yuan et al. [52] employed artificial intelligence for OCT image analysis, pushing the diagnostic accuracy further. Yang et al. [19] reported exceptional sensitivity and specificity of 98% and 99%, respectively, by utilising a texture-based analysis of OCT images. Yuan et al. [52] proposed a novel deep learning method, achieving an accuracy of 91.62%, with sensitivity and specificity of 91.66% and 92.58%, respectively, demonstrating the potential of AI in bridging the gap between training and testing OCT images for non-invasive oral cancer screening (Table 2).

The decision against performing a meta-analysis was also influenced by the variability in the reference standards used for confirming oral cancer diagnoses across the studies. Instead of a meta-analysis, a qualitative synthesis was conducted.

## 5. Expanded Insights

In the clinician-interpreted OCT category, Tsai et al. [59] utilised a swept-source OCT system, focusing on three key diagnostic indicators: standard deviation (SD) of the A-mode scan signal profile, the exponential decay constant (alpha), and epithelium thickness (T). Their findings highlight SD and alpha as reliable indicators for diagnosing moderate dysplasia and squamous cell carcinoma, showcasing OCT’s potential in differentiating between various oral pathological conditions based on tissue properties. Wilder-Smith et al. [60] demonstrated OCT’s excellence in detecting oral premalignancy and malignancy with high sensitivity and specificity, emphasising the method’s non-invasive nature and its ability to provide high-resolution imaging of the tissue subsurface, which is crucial for early diagnosis. Lee et al. [61] further validated OCT’s diagnostic accuracy, particularly in identifying moderate dysplasia from mild dysplasia lesions, through the use of standard deviation mapping, offering a quantitative approach to diagnosing oral precancerous conditions.

The algorithm-based OCT interpretations brought forth by Sunny et al. [28] and James et al. [62] introduced a transformative approach to oral cancer margin delineation and lesion detection. Sunny et al.’s study achieved a landmark sensitivity and specificity of 100%, underscoring the precision of an automated diagnostic algorithm in identifying malignancy within tumour margins. This represents a significant advancement in surgical outcomes, potentially reducing local recurrence rates. James et al.’s validation of a portable, algorithm-powered OCT device further illustrated the technology’s versatility and efficacy in a variety of clinical settings, highlighting its role in enhancing cancer screening and surveillance, especially in resource-constrained environments.

AI-driven OCT results mark a new frontier in oral cancer diagnosis. Yang et al. [19] and Yuan et al. [52] demonstrated the power of AI in analysing OCT images with unparalleled accuracy. Yang et al. [19] utilised texture-based analysis to achieve high diagnostic performance, emphasising the method’s potential in supporting surgeons with real-time, in vivo imaging for disease screening and diagnosis. Yuan et al.’s [52] introduction of the local residual adaptation network (LRAN) for non-invasive screening represents a significant leap towards harnessing deep learning for oral cancer diagnosis, showcasing the model’s ability to effectively bridge the gap between training and testing datasets, thus offering a promising solution to the challenges of manual biopsy screening.

These expanded insights underscore the evolving landscape of OCT technology in oral cancer detection, from clinician-based interpretations to cutting-edge AI analyses. The continuous advancements in OCT methodologies not only enhance diagnostic accuracy but also pave the way for more efficient, non-invasive screening techniques that could significantly impact patient care and treatment outcomes in oral oncology.

Finally, these studies [5,19,21,28,52,59,60,61,62] utilised a range of OCT systems, including swept-source OCT and handheld OCT devices, with some employing advanced algorithms and machine learning for improved diagnostic accuracy. Across these investigations, sensitivity and specificity varied but generally remained high, showcasing OCT’s effectiveness in distinguishing between normal, dysplastic, and malignant oral tissues. Notably, the incorporation of algorithm-based automated diagnostics and texture-based analysis further enhanced OCT’s capability to delineate oral lesions with high accuracy, highlighting its potential as both a screening and a diagnostic tool in the field of oral oncology. These findings underscore OCT’s significant promise in improving the early detection and management of oral cancer, thereby potentially improving patient outcomes (Table 3).

## 6. Discussion

By systematically reviewing the diagnostic capabilities of OCT and its integration with artificial intelligence (AI) and other imaging modalities, this research not only augments the existing body of knowledge but also charts a progressive course for future innovations in oral pathology diagnostics. This scientific rationale is well founded in the need to go beyond conventional diagnostic limitations through the use of the unparalleled resolution of OCT for real-time tissue morphology assessment. Moreover, it represents a leap towards the holistic integration of imaging technologies to boost early detection and precision in surgical intervention for oral cancer. This not only adds to the existing knowledge pool but also sets a new paradigm for innovation and research in diagnostics for oral healthcare, with an emphasis on the need to continually explore and validate clinical utility of OCT.

The great variation found in these studies among sensitivity and specificity underscores the pivotal role that technology development and operator expertise play in the effectiveness of OCT application. The first studies, although pioneering, represent an early phase in the use of OCT, with the major emphasis being laid on the capability of making a distinction between malignant and non-malignant tissues through visual or quantitative assessment by the physician [5,21,59,60,61]. As science and technology progressed, the incorporation of algorithm-based and AI-driven analyses into the field brought a new era of precision to oral cancer diagnostics. These improvements not only made the tests more accurate but also reduced the subjective variability attached to different interpretations of clinicians [19,28,52,62].

In this context, it can be noticed that later studies used machine learning algorithms and deep learning methods, significantly making OCT cross the gap between clinical examination and the need for histopathological confirmation [19,52]. This aspect makes way for a move towards personalised medicine, wherein diagnostic tools are progressively precise, reliable, and adapted to the profile of the individual patient.

However, the road from clinician-based to AI-fuelled OCT interpretation also underscores the standardisation obstacles these technologies face for widespread clinical application. Its diagnostic variability across studies thus points to the need for standardised protocols and calibration of OCT devices, ensuring consistency and reliability of results regardless of operating conditions or user expertise.

The integration of AI and machine learning not only holds promise for enhancing diagnostic accuracy but also for the identification of new biomarkers or tissue characteristics, pointing towards early malignant transformation. This could significantly impact all strategies in management related to oral cancer and make it possible for the condition to be caught earlier, with less morbidity and mortality from late diagnosis. Furthermore, incorporation of OCT with other diagnostic modalities can lead to a more comprehensive approach towards the diagnosis of oral cancer using morphological, functional, and molecular imaging techniques.

### 6.1. Advances in OCT Technology for Oral Pathology Assessment

Over the last decade, OCT has seen significant development in the field of oral pathology, and the quality and functionality of acquired OCT images have been greatly enhanced by advanced technologies. These improvements have dramatically increased the accuracy of diagnoses and completeness in the evaluation of the oral cavity. The recently developed swept-source OCT provides a greater scanning speed and higher tissue penetration for a comprehensive subsurface structure evaluation. Advanced imaging in the oral cavity provides high-resolution visualisation of oral tissues, thus allowing an accurate demonstration of microstructural changes within the oral structures. This forms the basis of early detection of pathologies, including oral cancer and periodontal disease, hence increasing possible prompt intervention [1,3,6,10,21,41,46,59].

In parallel with the structural improvements, the inclusion of functional imaging modalities in the OCT systems with angiography OCT and polarisation-sensitive OCT, clinicians have been provided tools for the visualisation of blood flow and assessment of tissue birefringence. This is critical for the assessment of vascular changes associated with malignancies and assessment of the collagen state and other structures in the oral cavity. Making access to this technology further democratised in dental practices, the proliferation of portable and handheld OCT devices takes diagnoses to the point of care and even closer when integrated with routine examinations. In addition, artificial intelligence for analysing OCT images makes it possible to detect and classify oral pathologies more simply and easily, leading to more accurate and consistent diagnoses. Telehealth functionalities allow OCT data to be shared and reviewed remotely so that expert analyses can be more widely available, and patients can have improved care regardless of location [8,9,12,13,19,52].

### 6.2. Advances in Image Enhancement Technology for Oral Pathology Assessment

The development of sophisticated image enhancement techniques applied to OCT images aimed at improving the quality, resolution, and interpretability of the OCT images considerably propelled the progress of OCT in oral pathology diagnosis. Among these kinds of enhancement, suppression of speckle noise is the interference in OCT images, which is primarily responsible for masking tissue details of importance for diagnostic purposes. Developed techniques for improved image clarity and contrast, with the preservation of tissue information integrity, include spatial compounding, wavelet-based methods, and adaptive filtering [7,8,15,62]. Besides, the improvement in the signal-to-noise ratio can be achieved by application of the technique of image averaging, which is further improved with the acquired capability of averaging of multiple OCT scans of the same region, to ensure ease in the early detection of subtle pathological changes by improving the definition of tissue boundaries [27,34].

Therefore, subsequent innovative extensions of traditional OCT, such as Doppler OCT and polarisation-sensitive OCT (PS-OCT), have added functional dimensions to OCT imaging. The ability of tissue to differentiate between benign and malignant lesions through the assessment of tissue vascularity and blood flow is a feature particularly beneficial in oral pathology [8,9,46]. The PS-OCT is based on the polarisation-changing properties of biological tissues, hence endowing an additional contrast mechanism in the images, valuable for structural integrity assessment. This is because the images are used to assess the collagen-rich tissue—common sites of oral pathologies [8,46]. In the meantime, breakthroughs in depth-enhanced technique development—such as EDI and swept-source OCT, alongside the integration of machine learning with OCT imaging—have revolutionised this field and led to several tools supporting rapid and correct diagnoses with deep tissue assessments, which considerably reduce the burden on operator expertise [1,5,22,62].

### 6.3. Integration with Other Imaging Modalities

The development of OCT for clinical use has advanced considerably through integration with other imaging modalities that can help to improve diagnosis and treatment of pathologies in the oral cavity. This integration will take advantage of the unique features that each imaging modality has to offer, for example, high-resolution features in OCT complementary to the visualisation of hard tissue provided by X-rays, CT scans, and MRI, allowing a more profound evaluation of both hard and soft tissues. Hence, the combination of these modalities, with proper results, is a fundamental requirement in the diagnostic process and in the treatment planning of complex cases, including but not limited to malignancies and advanced periodontal diseases [44]. In addition, the combination of OCT with other modalities, such as fluorescence imaging and Raman spectroscopy, greatly enhances the diagnostic potential by providing complementary information invaluable for carrying out a more integral assessment and making better-informed decisions in the field of oral pathology [31,35].

Other imaging advances include confocal and multiphoton microscopies, high-resolution modalities that offer the potential to use OCT concurrently with them in the oral cavity. The two modalities can, therefore, be combined to enhance each other and improve the ability of the clinician to make a diagnosis of cell or even subcellular pathology. For example, whereas OCT enables rapid image capture and a broader imaging scope, the use of confocal microscopy can zoom to the cell level, contributing to the crucial differentiation between benign and malignant lesions [46,61]. Indeed, the fusion of OCT with surgical navigation systems is another ground-breaking advancement in the discipline of oral and maxillofacial surgery. It offers real-time imaging feedback that directs the operating surgeon on safe and precise surgery. This integration is particularly valuable for tumour resection because it will allow a surgeon to excise the least amount of healthy tissue surrounding the tumour [1,21,22].

### 6.4. Challenges, Limitations, and Potential Solutions

OCT has become a tool that cannot be replaced in oral pathology, as it provides a large amount of information about subsurface structures and diagnosis. While holding all these advantages, the technical challenges of calibration, maintenance of the OCT systems, requirement of absolute alignment of optical components, and regular performance calibration clearly point towards the complexity associated with the use of this technology in a proper way. These are critical technicalities for the production of high-quality images, as even the smallest disparities result in a huge loss of diagnostic accuracy [47,50]. In addition, factors such as the SNR, which is affected by the saliva and blood of a patient and his/her movement, also play an important role in defining the quality of OCT images. These may lower the SNR, bring motion artifacts, and thus degrade the image quality, making the image unclear and unreliable for proper diagnosis [21,22,23].

OCT has a penetration depth of 2–3 mm at the maximum, and this poses another drawback, especially in the evaluation of deeper structures or pathologies. It will, therefore, lead to incomplete assessment in cases of diseases, such as periodontal disease, which requires the visualisation of much deeper tissues for a proper assessment [20,29]. In addition, because of the heterogeneity of oral tissues, the optical properties vary and will affect the performance of OCT. Tissue variations bring about differences in light absorption and scattering, thereby generating differences in contrast and image resolution, making it necessary to further understand the adjustments of imaging parameters to accommodate such differences for better interpretation and accurate information [21,34].

A basic characteristic that contributes strongly to the diagnostic potential of this imaging technique is the correlative relation between differences in the refractive indices of different oral tissues and quality and resolution in the OCT images. These differences in optical properties, such as the refractive index, result in varied light interactions and reflections at tissue interfaces, which are captured in contrast and resolution within OCT images. This would assist in increasing the contrast between tissues in an OCT image, a critical factor in boundary delineation and pathological change detection. Optimisation of these imaging parameters will, however, allow one to calibrate these refractive index differences in achieving improved OCT imaging capabilities for better discrimination between healthy and diseased oral tissues based on their optical signatures [1,21]. As a result, the accommodation of these refractive index variations not only enhances the precision and resolution of OCT images but also significantly increases its diagnostic potential in the identification and delineation of oral pathologies [22].

More importantly, the challenges reach far beyond technical and physical limitations and involve OCT image interpretation and operational expertise. Variability in the appearance of oral tissues among different individuals, combined with the dynamic nature of the oral cavity that never allows for complete immobilisation, emphasises the challenge of realising accurate and repeatable OCT images. These, coupled with the fact that larger pathologies require multiple scans and OCT usage is highly expensive, complex, and requires special training for optimum utilisation, further complicate its application in oral pathology [21,22,30,51,53]. However, ongoing research, including the one related to longer-wavelength light sources and machine learning algorithms, could overcome these burdens to make OCT more sensitive and more accessible for diagnostic purposes [7,11,33,34].

Innovations in OCT technology and in strategies to improve clinical utility, through higher tissue penetration, better image processing, and integration with other imaging modalities, continue unabated. Increased emphasis on training and education for users about the operation of OCT systems and the proper reading of images is also observed. The development of OCT in the field of oral pathology, with a special focus on these areas, envisages a lot of promise, and thus holds hope for developing a system that may potentially achieve more accurate diagnoses and improved patient results by means of relentless feedback from the medical community [33,34,60,61,62,63,64].

### 6.5. Future Directions

The future of OCT in oral pathology diagnostics and follow-up indeed looks bright and filled with several advances, as the technology is bound to get better in terms of image resolution, depth, ease of use, cost-effectiveness, and accessibility improvements [2]. Current trends to follow include Nano-OCT to achieve resolutions below the sub-micron level for visualising cellular and subcellular structures. The role of microscopic early detection/monitoring of oral diseases is, therefore, significant in changing the overall perspective on diagnostics of oral health [3,6].

The development of functional OCT is a step closer to providing not only an anatomical but also functional description of tissues with regard to blood flow, tissue elasticity, and molecular composition [9,11]. This new capacity for functional imaging is believed to enhance the study of oral pathologies, with the potential for more individually tailored and effective treatment strategies. At the same time, the efforts are ongoing to make the devices more portable and user-friendly, in such a way that OCT technology will be part of a routine practice in a dental setup, offering an on-the-spot availability of imaging tools used for quality assessment and diagnosis [13,63].

There is a gap between what research findings indicate in terms of potential for OCT to be used in the detection and monitoring of these oral pathologies and practical applicability in clinical settings. Hence, filling this gap would require intensive clinical trials to validate the outcomes of this research and standard protocols for the use of OCT in oral pathology assessments. Reliability and accuracy of OCT across different patient demographics and clinical environments are achieved when such policies result in practical ways of operationalising these aspects of OCT. It is also believed that OCT in routine dental visits will promise more accurate and individualised diagnostic services to all patients and, most importantly, to conditions such as oral cancer, where early detection improves outcomes [26,64].

In line with this, the OCT system is set to streamline patient care effectiveness and efficiency as it is aligned with clinical workflows [28]. The technology is non-invasive, and thus not only serves to better the patient experience but provides real-time imaging for immediate analysis and decision-making support. However, concerns relating to the expense of the equipment, training, and integration of OCT into the clinical workflow are necessary to achieve a smooth implementation in clinical practice. Future research involving the correlations of optical property changes in oral tissues with OCT imaging findings, along with an in-depth study on Nano-OCT technology, will further establish diagnostic accuracy and broaden the clinical applications of OCT in oral health [3,9,59].

## 7. Conclusions

Optical Coherence Tomography has made significant strides as a ground-breaking imaging technology in oral cancer diagnosis, showcasing a compelling blend of high-resolution capabilities and non-invasive imaging. This systematic review evaluated OCT’s diagnostic accuracy in detecting oral cancer and its progressive integration with other imaging modalities and artificial intelligence (AI), aiming to push the boundaries of early detection and treatment precision. The extensive analysis of selected studies revealed OCT’s remarkable effectiveness in distinguishing normal from pathological tissues, emphasising its pivotal role in early cancer detection and its substantial potential to enhance surgical intervention accuracy.

Future research should focus on harmonising OCT with various imaging technologies and the dynamic field of AI to further enhance its diagnostic precision and clinical utility. Such endeavours are crucial for maximising OCT’s role in improving oral health outcomes, calling for comprehensive, multicentric studies to thoroughly solidify its clinical relevance. OCT’s journey from clinician-based to AI-augmented interpretations underscores its transformative impact on oral cancer diagnostics, heralding a new era of efficiency and non-invasiveness in patient care within oral health diagnostics.

## Figures and Tables

**Table 1:** This table showcases the progression in OCT technology and its application in oral pathology.

Study Reference	OCT System and Approach	Key Findings	Clinical Importance
Tsai et al. [59], 2008	Swept-source OCT	Identified effective diagnostic indicators for oral cancer.	Enhances early detection and diagnosis of oral cancer.
Wilder-Smith [60] et al., 2009	In vivo OCT	Demonstrated high sensitivity and specificity in diagnosing oral dysplasia and malignancy.	Supports non-invasive diagnosis and monitoring of oral pathologies.
Lee et al. [61], 2012	OCT image analysis	Distinguished between normal and precancerous oral mucosae with high diagnostic statistics.	Aids in the differentiation of oral lesions with potential for precancerous development.
Hamdoon et al. [5], 2016	OCT for OSCC resection margins	Showed OCT’s value in assessing OSCC surgical margins.	Improves surgical outcomes by guiding resection margins.
Jerjes et al. [21], 2019	OCT for epithelial thickness	Found epithelial thickness measurement improves OCT’s cancer detection ability.	Enhances accuracy in identifying cancerous changes in oral tissues.
Sunny et al. [28], 2019	Intra-operative OCT	Demonstrated OCT’s high sensitivity and specificity in delineating oral cancer margins.	Facilitates precise surgical margin assessment during oral cancer surgery.
James et al. [62], 2021	Portable OCT with machine learning	Validated a point-of-care OCT device for detecting oral potentially malignant and malignant lesions.	Offers a non-invasive tool for oral cancer screening and surveillance.
Yang et al. [19], 2022	Swept-source OCT with texture analysis	Achieved high accuracy in identifying oral precancerous and cancerous tissues.	Enhances the diagnostic process through detailed tissue characterisation.
Yuan et al. [52], 2022	OCT with deep learning for screening	Proposed a novel deep learning method for non-invasive oral cancer screening.	Streamlines oral cancer diagnosis with high accuracy and non-invasiveness.

**Table 2:** Summary of recent clinical trials using OCT for assessment of oral malignancy.

Study Reference	OCT		Control		Sensitivity	Specificity	Image Interpretation
	Event	Total	Event	Total			
Tsai et al. [59], 2008	21	36	7	43	75%	71%	Clinician
Wilder-Smith et al. [60], 2009	32	33	3	17	91%	93%	Clinician
Lee et al. [61], 2012	36	45	8	92	82%	90%	Clinician
Hamdoon et al. [5], 2016	72	84	6	41	92%	74%	Clinician
Jerjes et al. [21], 2019	46	65	5	174	90%	90%	Clinician
Sunny et al. [28], 2019	24	24	0	23	100%	100%	Algorithm-based
James et al. [62], 2021	91	96	5	21	95%	76%	Algorithm-based
Yang et al. [19], 2022	416	421	8	525	98%	99%	AI
Yuan et al. [52], 2022	132	141	12	123	92%	92%	AI

**Table 3:** This table outlines the main limitations identified within each study and suggests future directions that could address these challenges, thereby advancing the field of OCT-based oral cancer diagnosis.

Study	Limitations	Future Directions
Tsai et al. [59], 2008	Limited by the sample size and the specificity in distinguishing between different types of dysplasia.	Expand sample size and refine diagnostic criteria for varying dysplasia grades.
Wilder-Smith et al. [60], 2009	Study was preliminary and involved a small cohort of patients.	Conduct larger-scale studies to validate findings and improve diagnostic algorithms.
Lee et al. [61], 2012	Focus was primarily on moderate dysplasia, with less emphasis on severe cases.	Include a wider range of dysplasia severity to enhance diagnostic applicability.
Hamdoon et al. [5], 2016	Relied on ex vivo analysis of resection margins, which may not fully replicate in vivo conditions.	Develop techniques for in vivo margin assessment to guide real-time surgical decisions.
Jerjes W al. [21], 2019	The effect of tissue processing on OCT measurements was noted, potentially affecting accuracy.	Investigate methods to account for tissue-processing effects and improve measurement consistency.
Sunny et al. [28], 2019	Focused on a relatively small number of patients and surgical sites.	Increase the number of participants and sites for a more comprehensive evaluation.
James et al. [62], 2021	Utilised machine learning algorithms that require extensive validation across diverse populations.	Test and refine algorithms in varied demographic and clinical settings to ensure generalisability.
Yang et al. [19], 2022	Limited by the study’s reliance on ex vivo tissue samples.	Advance towards real-time, in vivo diagnostic applications to enhance clinical utility.
Yuan et al. [52], 2022	Depended on a novel deep learning method that may require further optimisation.	Continue to refine and test the deep learning framework on larger datasets to validate efficacy.
